# Chronic Cystitis—Drainage

**Published:** 1874-08

**Authors:** Hunter McGuire


*RICHMOND & LOUISVILLE MEDICAL JOURNAL*—*June*, 1874.

Drainage in Obstinate Chronic Inflammation of the Bladder. *By Hunter McGuire, M.D.*—Mrs. Blank, aged forty-one years, came to me about the first of July, 1873, laboring under chronic inflammation and perhaps ulceration of the bladder. The disease came on about eight years ago, she thinks, in consequence of exposure to cold. * * * I had on my office table, when she came in, a piece of gum tubing, made of the finest gum, about twelve inches long, and four inches of it, or that portion which was to be placed in the bladder, had been perforated by a shoemaker's punch, with holes about a half an inch apart. * * * The silver tube was introduced very carefully through the urethra into the bladder, and the gum tube pushed through this until four or five inches had been coiled up in the bladder. The silver tube was then withdrawn, leaving the gum tube in the bladder. Two pieces of tape were then fastened to the gum tube by a solution of gum-elastic in chloroform, and these tapes tied to a band which had been passed around the woman's hips. The drainage tube being thus secured in the bladder, the free end of the tube was put into a common bottle to catch the urine. * *

The lady informed me the next morning that she had passed a more comfortable night than she had done for years. The drainage was kept up without interruption until about daylight, when the holes became filled with mucus, the flow ceased and the tube was expelled by the bladder. I re-introduced it, and directed the nurse to examine the tube every half hour, and if it became clogged, to inject half an ounce of tepid water through it, and in this way keep it open. * * *

For six weeks this tube was kept in the bladder constantly, and the patient remained in bed. Frequently it was expelled by the bladder, and required to be replaced, and very often new tubing was substituted for the old. I found the shorter the portion of tubing in the bladder, the longer it would be retained, and at last introduced only enough to secure the drainage; but the bladder evidently became more and more tolerant of the presence of the foreign body after the first week had passed. * * * Occasionally phosphatic deposits were found on the tube, but this was corrected by the use of citric acid in the shape of lemonade. During this period she slept well, was free from pain, her appetite increased, and her general health rapidly improved.

At the end of six weeks I attached the free end of the tube to a gum bag, which was fastened to the patient's thigh just above the knee. She was then permitted to get up and go about the house, and after a few days, to walk about. She wore the tube for four months, going about and attending to many of her household duties, and enjoying a freedom from pain and a degree of comfort which she had not felt for years. At the end of this time I had some difficulty in persuading her to let me remove the tubing, so much afraid was she of a return to her former suffering. There was for some time after its removal complete incontinence of urine; but after a few days, especially after lying down, the bladder began gradually to retain some urine, and to expel it in small quantities. The organ gradually recovered its former healthy power, and at this time, eight months since the drainage was begun, she can retain the urine about three hours, and void it without pain. Except the slightly increased frequency of micturition—and this is growing less—the lady is well.

